# The Associations of Electronic Health Record Usability and User Age With Stress and Cognitive Failures Among Finnish Registered Nurses: Cross-Sectional Study

**DOI:** 10.2196/23623

**Published:** 2020-11-18

**Authors:** Anu-Marja Kaihlanen, Kia Gluschkoff, Hannele Hyppönen, Johanna Kaipio, Sampsa Puttonen, Tuulikki Vehko, Kaija Saranto, Liisa Karhe, Tarja Heponiemi

**Affiliations:** 1 Finnish Institute for Health and Welfare Helsinki Finland; 2 Department of Computer Science Aalto University Helsinki Finland; 3 Finnish Institute of Occupational Health Helsinki Finland; 4 Department of Health and Social Management University of Eastern Finland Kuopio Finland; 5 Finnish Nurses Association Helsinki Finland

**Keywords:** electronic health records, usability, stress, cognitive failure, nurse

## Abstract

**Background:**

Electronic health records (EHRs) are expected to provide many clinical and organizational benefits. Simultaneously, the end users may face unintended consequences, such as stress and increased cognitive workload, due to poor EHR usability. However, whether the effects of usability depend on end user characteristics, such as career stage or age, remains poorly understood.

**Objective:**

The objective of this study was to examine the associations of EHR usability and user age with stress related to information systems and cognitive failures among registered nurses.

**Methods:**

A cross-sectional survey design was employed in Finland in 2017. A total of 3383 registered nurses responded to the nationwide electronic survey. Multiple linear regression was used to examine the associations of EHR usability (eg, how easily information can be found and a patient’s care can be documented) and user age with stress related to information systems and cognitive failures. Interaction effects of EHR usability and age were also tested. Models were adjusted for gender and employment sector.

**Results:**

Poor EHR usability was associated with higher levels of stress related to information systems (β=.38; *P*<.001). The strength of the association did not depend on user age. Poor EHR usability was also associated with higher levels of cognitive failures (β=.28; *P*<.001). There was a significant interaction effect between age and EHR usability for cognitive failures (β=.04; *P*<.001). Young nurses who found the EHR difficult to use reported the most cognitive failures.

**Conclusions:**

Information system stress due to poor EHR usability afflicts younger and older nurses alike. However, younger nurses starting their careers may be more cognitively burdened if they find EHR systems difficult to use compared to older nurses. Adequate support in using the EHRs may be particularly important to young registered nurses, who have a lot to learn and adopt in their early years of practice.

## Introduction

Electronic health record (EHR) systems have increasingly replaced paper-based practices in hospitals, with the expectation of providing many clinical and organizational benefits [1,2]. Implementation of EHRs will inevitably change work practices in health care [3,4] and, if not properly managed, may result in many unexpected and unintended consequences. In addition to the benefits identified (eg, reduction of medication errors), health care professionals have reported disadvantages, such as increased emotional strain and increased errors when trying to learn and adapt to new technologies and managing their workflow disruptions [2,5,6]. Implementation and use of EHRs have also required increased effort from professionals in performing their typical task flow [7], which in turn has resulted in increased cognitive workload and decreased cognitive performance [8]. In addition to missing focus on proactive workflow redesign during EHR implementation, earlier studies have found that many of the unfavorable consequences are connected with the usability issues of EHR systems, referring to how easy the system is to use and how precisely and efficiently required tasks can be performed [9,10].

Nurses, as the largest group of health care professionals, are the main end users of the EHRs, and their daily work is greatly influenced by ease of use as well as technical and functional quality of the systems. According to previous studies, both Finnish nurses and physicians are dissatisfied with the usability of their EHR systems [11]. Nurses’ experiences of the poor usability of EHRs and how they can negatively affect workflow appear to be consistent across countries [12,13]. Ease of use and high quality of information systems promote the use of technology and support the management of care records [14]. However, EHRs that are perceived as difficult to use have been shown to be associated with increased stress levels [15] and cognitive workload among nurses [16,17], which may consequently increase the risk of cognitive failures [18]. Cognitive failure is defined as “a cognitively based error that occurs during the performance of a task that a person is normally successful in executing” [19]. The failure can occur in a person’s memory functions, attention regulation, or actions [20], and the incidence is connected with work environment–related [18,21] and individual factors [20].

So far, only limited and contradictory knowledge exists on whether the consequences of poor EHR usability, particularly stress or impairment in cognitive functions, could depend on end user characteristics such as age. First, age-group differences in the likelihood of experiencing EHR-related stress have not been found [22]. However, experienced (and thus likely older) nurses can have more negative attitudes toward the use of new technologies in clinical work [23]. They may potentially experience more stress and higher cognitive workload than younger professionals, who may adapt better to digitization-related changes [16]. Second, youth has been associated with higher nursing informatics competence, such as skills in electronic documentation and use of information technology [24], which can make working with the EHRs easier and be a factor in protecting nurses’ well-being at work [15]. Nevertheless, while young nurses starting their careers may be more skilled in and used to using technology than older nurses, they are still probably less experienced in using EHR systems in their work. Multiple studies have suggested that integrating EHRs into daily workflow and performing EHR-related tasks may be easier and require less cognitive effort from more experienced EHR users than from novice users [17,25].

Based on our knowledge, there is little evidence of whether nurses of a certain age are more at risk of experiencing stress or increased cognitive workload due to poor usability of EHR systems. In light of previous evidence, differences may exist between nurses of different ages, but the findings are mixed and the potential moderating effect of EHR usability has not been investigated. Moreover, previous studies examining the negative outcomes linked to EHR usability, such as stress, have mainly focused on physicians [26-28] and less on nurses [22]. Identifying those most at risk of experiencing EHR-related disadvantages is important in order to provide them with adequate support in using the systems. Most importantly, the topic requires further investigation because problems related to EHR usability and subsequent issues of stress and impairment in cognitive performance are notable threats to the quality of care and patient safety [18,29,30]. This study aimed to investigate the associations of EHR usability and user’s age with stress related to information systems and cognitive failure at work among registered nurses. Additionally, we examined whether the possible associations of EHR usability with stress related to information systems and cognitive failures are modified by user age.

## Methods

### Setting, Data Collection, and Participants

In 2017, a nationwide cross-sectional survey was conducted in Finland on registered nurses’ experiences with currently used EHR systems [15,31,32]. The data were collected with a web-based questionnaire that was sent to all the registered nurses (n=29,283) who were members of the Finnish Nurses Association and the National Association of Health and Welfare Professionals and had provided an email address. Altogether, 3607 of the 29,283 nurses responded to the questionnaire (a 12.3% response rate). Nurses with missing information on any of the demographic variables (age, gender, employment sector) were excluded from the study (n=224), resulting in a final sample of 3383 nurses. In the Finnish public health care system, the EHR coverage has been 100% since 2010 [33]. Over 20 EHR brands are being used in different health and social care settings [31].

### Measurements

The EHR usability was measured with 7 ease of use–related items (α=.84) from the validated National Usability-Focused Health Information System Scale (NuHISS) [34]. The items were as follows: (1) the arrangement of fields and functions is logical on a computer screen; (2) the systems keep me clearly informed about what it is doing (eg, saving data); (3) terminology on the screen is clear and understandable (eg, titles and labels); (4) routine tasks can be performed in a straightforward manner without the need for extra steps using the system; (5) it is easy to obtain necessary patient information using the information system; (6) entering and documenting patient data is quick, easy, and smooth; and (7) the information on the nursing record is in an easily readable format. Items were rated on a 5-point scale (1=fully disagree to 5=fully agree). A higher score on ease of use items indicates better experienced usability.

Stress related to information systems was measured with 2 items (α=.62) that evaluated how often during the past half-year period the person has been distracted, worried, or stressed about (1) constantly changing information systems and (2) difficult, poorly performing information technology equipment or software [26]. The items were rated on a 5-point scale ranging from 1 (never) to 5 (very often). This measure has been previously used with physicians and is associated with EHR usability and distress [27,35].

Cognitive failures were measured with 3 items (α=.59) modified from the 15-item Workplace Cognitive Failure Scale (WCFS) [20,36]. The WCFS includes 3 dimensions: failure in memory, failure in attention, and failure in action. Regarding the length of the survey questionnaire, we had to limit the number of questions and chose 1 item per dimension to measure nurses’ cognitive failures. The selection of these items was based on their highest loadings for the 3 factors or dimensions of cognitive failure [20]. Participants were asked to rate how often they have faced situations at work where they (1) have not remembered a work-related password, set of numbers, etc (memory failure); (2) have not fully listened to the instructions or requests they have received (attention failure); or (3) have accidentally started or closed the wrong device, system, or program (action failure). Items were answered on a 5-point scale ranging from 1 (never) to 5 (several times a day).

Other variables included were age, gender, and employment sector (1=hospital, 2=health center, 3=private sector, 4=social services, or 5=other).

### Data Analysis

Continuous variables are summarized using mean and standard deviation, and categorical variables are presented as the number of participants and percentage. Multiple linear regression was used to examine the associations of EHR usability and nurse’s age with stress related to information systems and cognitive failures. This method was chosen because the associations were assumed to be linear, and it offered easily interpreted output coefficients and a less complex algorithm compared to many other methods. Analyses were conducted separately for both dependent variables (stress related to information systems and cognitive failures). In the first step, EHR usability and age were included as predictors in the model. In the second step, the combined effect of EHR usability and age was tested by adding an interaction term to the former model. Age was divided by 10 for the analysis to assess a given decade’s association and make the estimated coefficients easier to interpret. All models were adjusted for gender and employment sector. The analyses were conducted using RStudio.

## Results

### Characteristics of the Participants

The majority of the participants were female (3204/3383, 94.7%). They were, on average, 46.2 years old (range: 22-66), and over half of the participants worked in hospitals. There were differences in the estimated EHR usability (*P*<.001), stress related to information systems (*P*<.001), and cognitive failures (*P*=.02) between nurses working in different work environments. The EHR usability was rated highest among nurses who worked in social services. Nurses working in hospitals gave the lowest EHR usability ratings and had more stress related to information systems than nurses working in other fields. There were no gender differences in the values of the variables studied. Characteristics of the participants and descriptive statistics of the study variables are presented in Table 1.

**Table 1:** Characteristics of the participants (N=3383) and descriptive statistics.

Characteristic		n (%)		Mean		SD		Minimum		Median		Maximum	
Age, years		N/A^a^		46.22		11.1		22		48		66	
**Gender**													
	Male		169 (5.0)		N/A		N/A		N/A		N/A		N/A
	Female		3204 (94.7)		N/A		N/A		N/A		N/A		N/A
	Other		10 (0.3)		N/A		N/A		N/A		N/A		N/A
**Employment sector**													
	Hospital		1796 (53.1)		N/A		N/A		N/A		N/A		N/A
	Health center		707 (20.9)		N/A		N/A		N/A		N/A		N/A
	Private clinic		173 (5.1)		N/A		N/A		N/A		N/A		N/A
	Social services		433 (12.8)		N/A		N/A		N/A		N/A		N/A
	Other		274 (8.1)		N/A		N/A		N/A		N/A		N/A
Usability		N/A		3.04		0.78		1		3		5	
SRIS^b^		N/A		3.02		0.89		1		3		5	
Cognitive failures		N/A		1.96		0.51		1		2		5	

^a^N/A: not applicable.

^b^SRIS: stress related to information systems.

### Associations of EHR Usability and User Age With Stress Related to Information Systems and Cognitive Failures

The results of the linear regression analyses are shown in Table 2. The EHR usability was associated with both stress related to information systems (β=.38; *P*<.001) and cognitive failures (β=.28; *P*<.001). Higher levels of usability were associated with lower levels of both stress related to information systems and cognitive failures. Age was associated with cognitive failures (β=.16; *P*<.001) but not with stress related to information systems. Younger nurses had higher levels of cognitive failure compared to older nurses.

**Figure 1:**
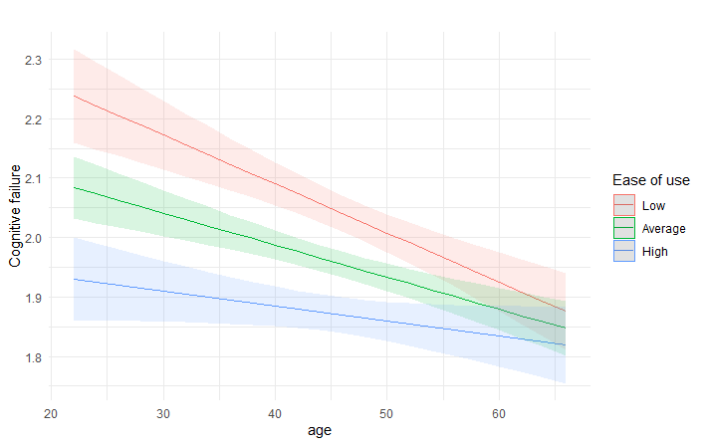
Interaction effect between EHR usability (ease of use) and user’s age for cognitive failures. The association is shown for low (mean − 1 SD), average, and high (mean + 1 SD) levels of ease of use. EHR: electronic health record.

**Figure 2:**
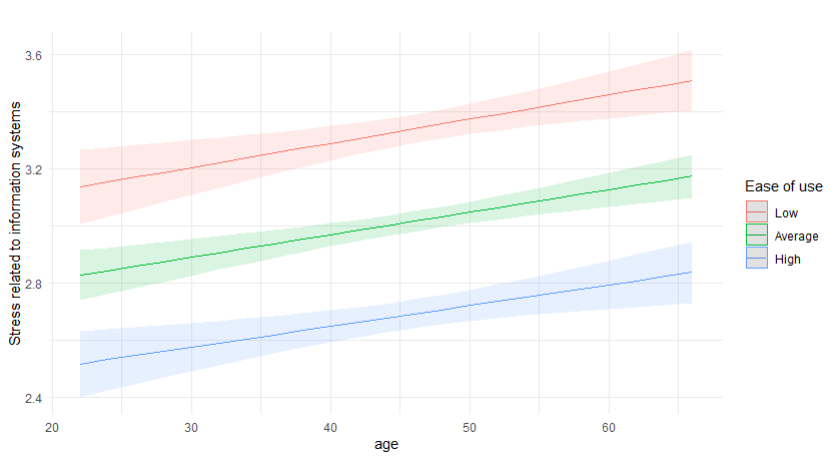
Interaction effect between EHR usability (ease of use) and user’s age for information system–related stress. The association is shown for low (mean − 1 SD), average, and high (mean + 1 SD) levels of ease of use. EHR: electronic health record.

There was a significant interaction effect between age and EHR usability for the cognitive failures (β=.04; *P*<.001). Younger nurses who evaluated the EHR as difficult to use had the highest levels of cognitive failures. Among older nurses, usability was not associated with their cognitive failure levels (Figure 1). There was no interaction effect between age and EHR usability for the stress related to information systems (Figure 2). Figure 1 and Figure 2 illustrate the interaction effects.

**Table 2:** The associations of age and EHR usability with stress related to information systems and cognitive failures.

Variable			Estimate	*P* value
**SRIS^a^**				
	Age		.10	.15
	Gender		.13	.10
	**Employment sector**			
		Hospital	Reference	N/A^b^
		Health center	.10	.03
		Private clinic	.32	<.001
		Social service	.18	<.001
		Other	.16	.03
	Usability		.38	<.001
	Age × usability		.01	.80
	Adjusted R^2^		0.16	N/A
**Cognitive failures**				
	Age		.16	<.001
	Gender		.02	.74
	**Employment sector**			
		Hospital	Reference	N/A
		Health center	.08	.01
		Private clinic	.03	.60
		Social service	.00	.93
		Other	.04	.33
	Usability		.28	<.001
	Age × usability		.04	<.001
	Adjusted R^2^		0.04	N/A

^a^SRIS: stress related to information systems.

^b^N/A: not applicable.

## Discussion

### Principal Findings

This study examined the associations of EHR usability and user’s age with stress related to information systems and cognitive failures among Finnish registered nurses. The practical goal was to increase system vendors’, health care managers’, and nursing educators’ awareness of the potential consequences of poor EHR usability, a shared problem among nurses in many countries that may jeopardize the quality and safety of care [12,29]. As predicted, we found that EHR usability was associated with both stress related to information systems and cognitive failures. Nurses who provided higher usability ratings had less stress and fewer cognitive failures compared to nurses providing lower ratings. The nurses’ age, in turn, was not associated with stress related to information systems, but it was negatively associated with cognitive failures. We also found a significant interaction effect between age and EHR usability for cognitive failures, indicating that young nurses who rated EHRs as difficult to use had the most cognitive failures.

The finding that young nurses’ cognitive functions may be more impaired by poor EHR system usability compared to those of older nurses seems logical. The youngest nurses have probably worked in the field for the shortest time, and the early stages of a nursing career are known to be demanding and challenging, which can lead to their attentiveness and memory being strained [37,38]. In addition, older nurses with more experience of using EHRs and various EHR brands may find it easier to integrate information systems into daily workflow than less experienced nurses [25]. A previous study has shown conflicting results and found a relationship between higher age and increased risk of cognitive failures among nurses [21]. However, this result was explained by the fact that older nurses may have lower work ability, which is associated with an increased risk of cognitive failures in certain work environments [21]. Differences in work environments also emerged in this study, and it appears that EHRs may be particularly burdensome for nurses working in hospital settings, who found the systems most difficult to use and experienced the most stress compared to nurses working in other environments. It would be important to find out the views of nurses working in the hospital environment about the weaknesses of EHRs and how systems should be developed to improve their perceived usability and thereby reduce stress.

Due to new role adjustment, duties, responsibilities, and work environments, many new nurses experience increased stress and emotional exhaustion [39]. These symptoms have been associated with deterioration in cognitive performance (eg, in attention and memory functions), and this applies especially to professions with high levels of work pressure and intense cognitive demands, like nursing [40]. Although we only looked at stress related to information systems in this study, it is possible that the high level of strain early in their careers may partly explain why young nurses in this study had more cognitive failures. Moreover, due to lack of expertise and the fact that the youngest generation of nurses are the most likely to change jobs [41,42], they constantly have much to learn at work. This, on top of a load caused by EHRs that are difficult to use, may increase task stressors, such as performance constraints, task uncertainty, or difficulties managing time pressure and frequent interruptions, all of which are shown to foster cognitive failure [43].

It is evident that EHRs should support nurses in carrying out their work tasks and not in turn increase workload, stress, or cognitive burden. A recent review by Wisner et al concluded that EHRs have the potential to support the cognitive work of health care staff, but the scattering of information, information complexity, and lack of chronology often hampers this. Encountering problems while trying to find or synthesize information can affect a nurse’s ability to achieve and maintain clinical understanding and situational awareness, which can compromise patient safety [8]. Usability and stability of information systems as well as end user involvement in system development and work procedure planning may be significant factors in alleviating stress related to information systems [15,26]. Since improving the usability of EHR systems seems to be challenging, the importance of adequate orientation and support at work to use information systems is critical.

In this study, challenges were observed especially in young nurses, whereby there is also a need to discuss whether current nursing education provides students with adequate knowledge and skills on how to use and integrate EHRs into daily work. Shortcomings have been identified in both theoretical and practical studies and, for example, in students’ opportunities to practice documentation with real EHR systems during their education [44,45]. The fact that using the systems can often only be learned and practiced in the workplace after graduation puts an additional burden on young nurses when there is still a lack of mastery of the work and nursing as a whole. Moreover, the large number of different EHR brands and their differences in usage logic, for example, may slow the process of learning to use them.

Currently, work tasks that require the use of EHRs, such as documentation, take up a significant portion of nurses’ day-to-day working hours [46]. Potential time pressure can be alleviated by having a high-quality information system [15]. Our study suggests that young nurses in particular could benefit from well-designed and implemented EHR systems that support routine tasks (eg, easy access to the information needed to treat the patient). Another interesting finding was that while poor usability of EHRs was associated with nurses’ higher stress related to information systems, the level of stress did not vary significantly between younger and older nurses. In other words, the levels of tolerance of EHR usability problems appeared to be equal in nurses of different ages. The results of this study contradict the stereotypical idea that millennial nurses who have grown up with digitization and who are more accustomed to coping with a variety of electronic platforms and tasks simultaneously [47] would automatically be less burdened by information systems than those nurses who have had to learn to work with them at a later age. Older nurses may compensate for the slower adoption of information systems with their experience and better management of patients’ overall care.

### Limitations

Possible limitations of this study should be considered when interpreting the results. First, in spite of representativeness of the responses in a large sample of Finnish registered nurses [31], the response rate to the survey remained rather low. This may limit the generalizability of the findings to a larger research population. Second, we were able to use only 3 items from the scale measuring cognitive failures (WCFS), and the reliability of this measure (0.59) can be considered low. The WCFS has demonstrated high internal consistency for the whole scale and subscale level and the 3 items that were chosen for this study are the most indicative of the 3 components (attention, memory, function) of cognitive failure [20]. Third, although we controlled the analysis for gender and employment sector, we are aware that some other variables may have contributed to stress related to information systems and cognitive failures as well (such as how long a person has used the current EHR system). Finally, the cross-sectional design did not allow the detection of causal relationships of the variables under study. The data used in this study was based on the first national survey of Finnish nurses gathered using the validated NuHISS [34]. A resurvey will be conducted in 2020, which will allow for further investigation of this topic.

### Conclusions

Poor usability of EHRs can place a significant strain on the day-to-day work of nurses. This study suggests that cognitive performance, especially among young nurses, may be disturbed due to poor EHR usability. Young nurses need support and familiarization in many aspects of nursing during their first years in practice, and attention should be paid to providing them with appropriate support and training in the use of EHRs, which takes up a considerable amount of their working time. The results indicate that young nurses, who are typically believed to be fluent information technology users, may be burdened with poorly functioning information systems, possibly even more than their older colleagues are. System vendors have the primary responsibility to ensure the usability of their systems and to contribute to the quality of care and patient safety. It could be useful to investigate whether some usability factors are more critical than others. However, addressing the weaknesses of EHRs may be slow. In order to tackle the adverse consequences, it is important that employers provide adequate support for the right groups and that educational institutions provide students with adequate training in the use of EHRs. Further research should pay attention to the experiences of nurses of different ages and at different career stages in relation to the use of EHRs. It would also be useful to investigate the relationship between EHR education received as a student and early career stress and cognitive burden related to information systems. Finally, studies with longitudinal designs are needed to detect causal associations such as whether usability problems lead to cognitive failures.

## Abbreviations

EHR: electronic health record
NuHISS: National Usability-Focused Health Information System Scale
WCFS: Workplace Cognitive Failure Scale
